# Supplementary material for: A Humanized Yeast Phenomic Model of Deoxycytidine Kinase to Predict Genetic Buffering of Nucleoside Analog Cytotoxicity
Source: Genes (Basel). 2019 Sep 30;10(10):770. doi: 10.3390/genes10100770 (PMC6826991; doi:10.3390/genes10100770)
Supplement: Supplementary file 1 [file genes-10-00770-s001.zip › Additional_Files/Additional_File7_GOTermHeatmaps.zip!/Additional_File7_GOTermHeatmaps/vesicle_docking.pdf]

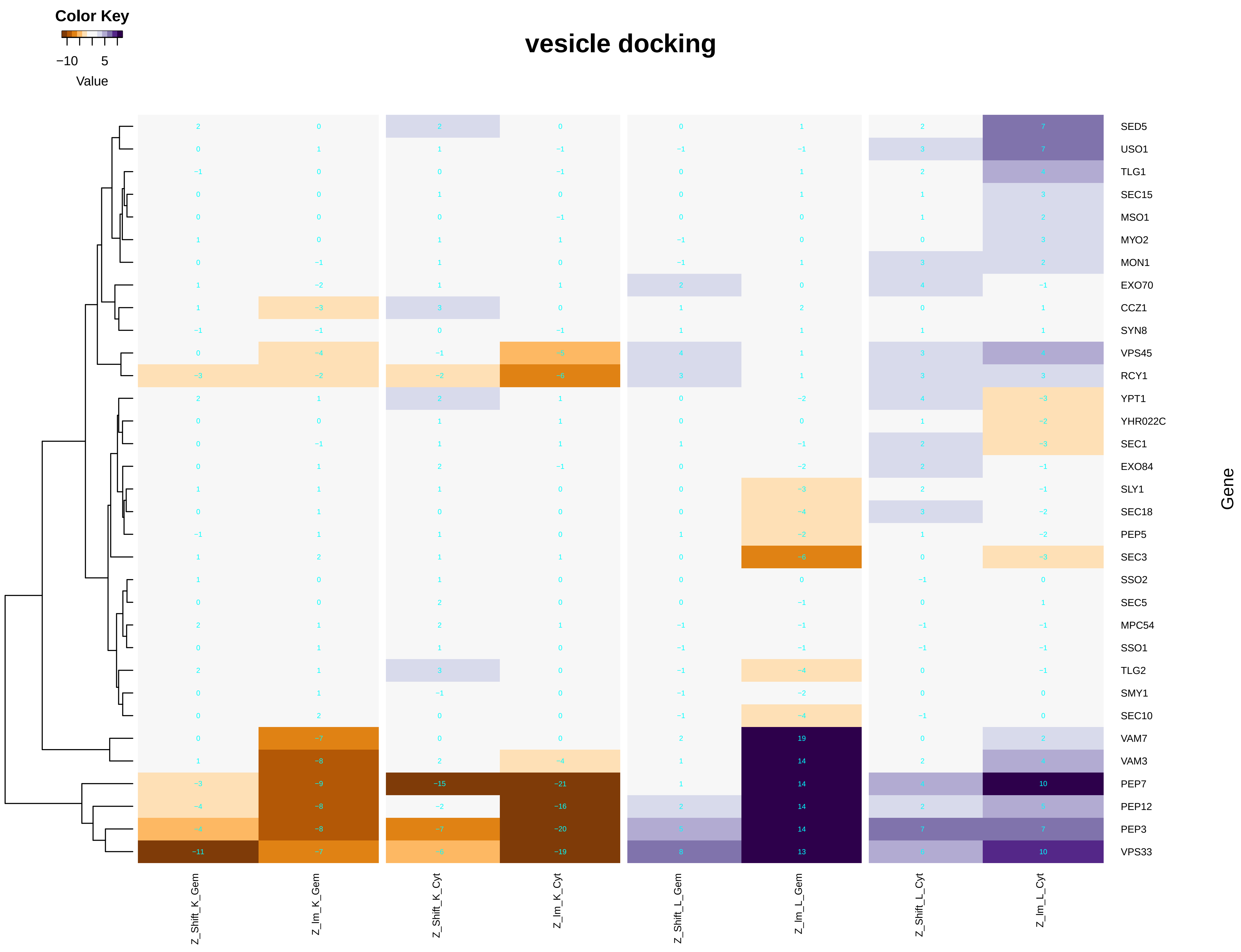
Color Key
−10 5
Value
vesicle docking
SED5
USO1
TLG1
SEC15
MSO1
MYO2
MON1
EXO70
CCZ1
SYN8
VPS45
RCY1
YPT1
YHR022C
SEC1
EXO84
SLY1
SEC18
PEP5
SEC3
SSO2
SEC5
MPC54
SSO1
TLG2
SMY1
SEC10
VAM7
VAM3
PEP7
PEP12
PEP3
VPS33
Gene
Z_Shift_K_Gem
Z_Im_K_Gem
Z_Shift_K_Cyt
Z_Im_K_Cyt
Z_Shift_L_Gem
Z_Im_L_Gem
Z_Shift_L_Cyt
Z_Im_L_Cyt

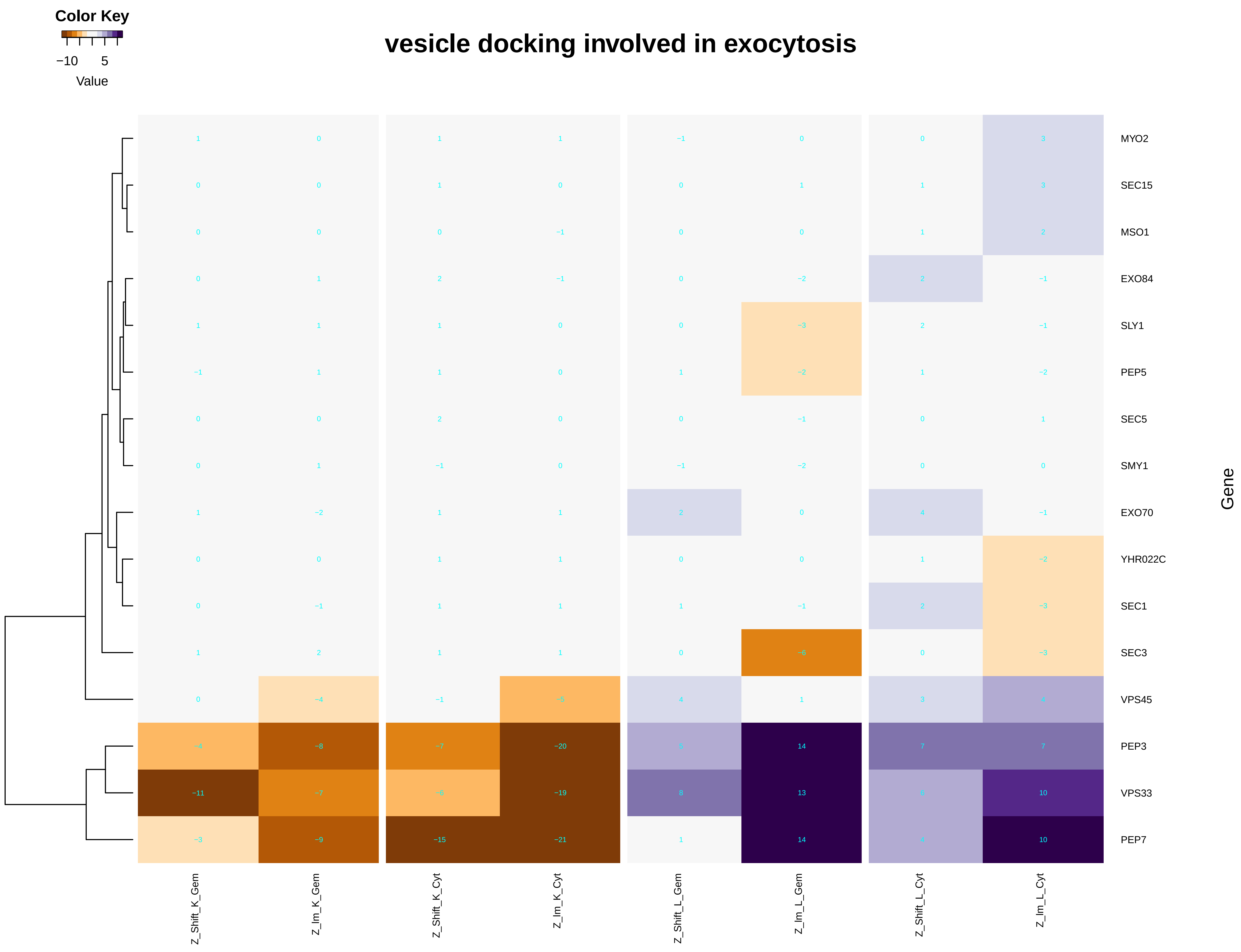

Color Key
−10
5
Value
vesicle docking involved in exocytosis
MYO2
SEC15
MSO1
EXO84
SLY1
PEP5
SEC5
SMY1
EXO70
YHR022C
SEC1
SEC3
VPS45
PEP3
VPS33
PEP7
Gene
Z_Shift_K_Gem
Z_Im_K_Gem
Z_Shift_K_Cyt
Z_Im_K_Cyt
Z_Shift_L_Gem
Z_Im_L_Gem
Z_Shift_L_Cyt
Z_Im_L_Cyt

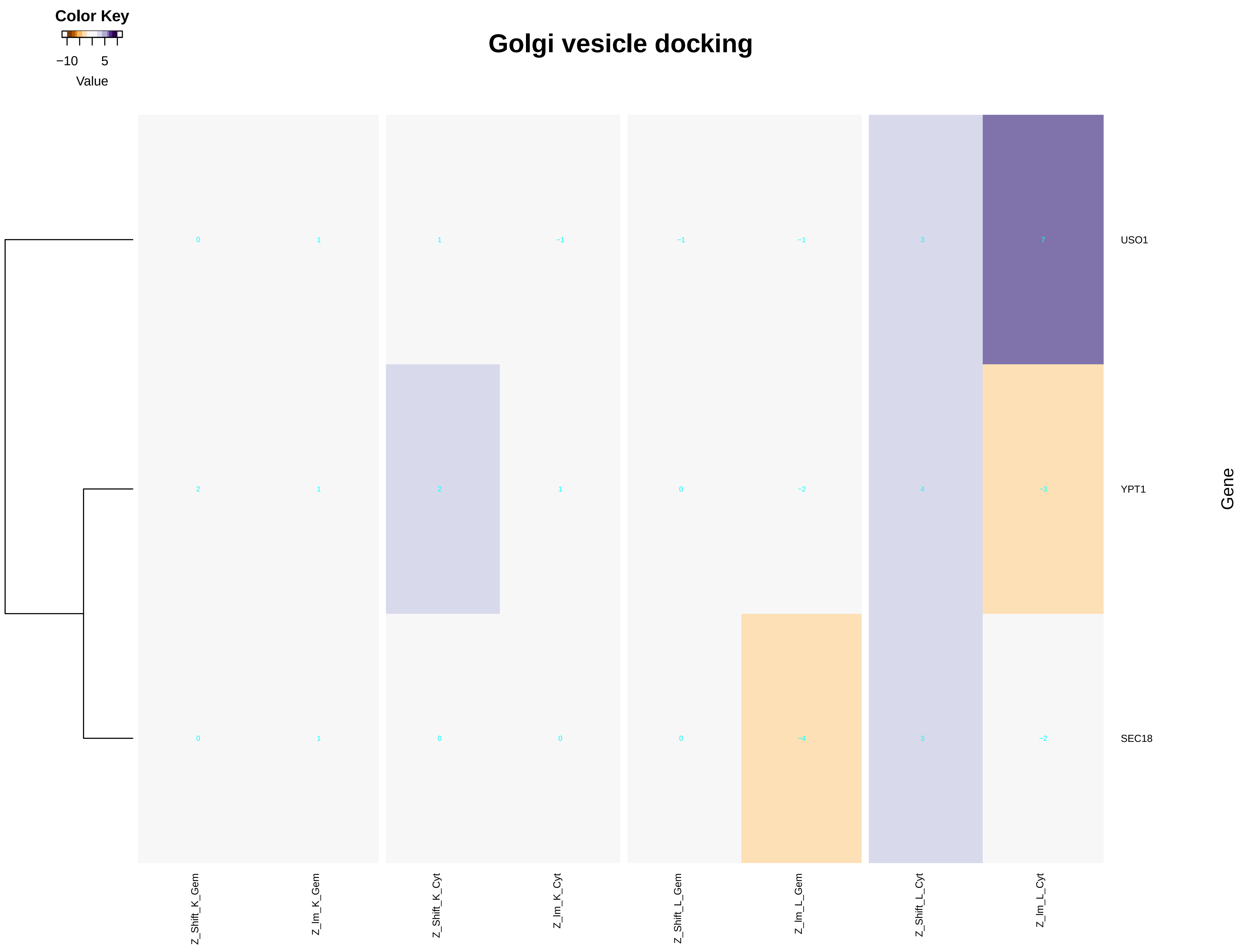
Color Key
−10
5
Value
Golgi vesicle docking
USO1
YPT1
SEC18
Gene
Z_Shift_K_Gem
Z_Im_K_Gem
Z_Shift_K_Cyt
Z_Im_K_Cyt
Z_Shift_L_Gem
Z_Im_L_Gem
Z_Shift_L_Cyt
Z_Im_L_Cyt
